# Oxidative DNA Damage as an Integrative Marker of Redox Dysfunction Associated with Doxorubicin-Induced Cardiotoxicity in Pediatric Leukemia

**DOI:** 10.3390/cimb48060577

**Published:** 2026-06-01

**Authors:** Jesús Alonso Gándara-Mireles, Elio Aarón Reyes Espinoza, Verónica Loera-Castañeda, Lourdes Patricia Córdova Hurtado, Antonio Emilio González Font, Julio Cesar Grijalva Ávila, Ignacio Villanueva Fierro, Ismael Lares-Asseff, Cynthia Mora Muñoz, Gabriela Velasco Villa, Hugo Payán Gándara, Leslie Patrón-Romero, Horacio Almanza-Reyes

**Affiliations:** 1 CIIDIR-Durango Unit, National Polytechnic Institute, Genomics Academy, Durango 34220, Mexico; jcgrijalva69@gmail.com (J.C.G.Á.); ivillanuevaf@ipn.mx (I.V.F.); 2 Latin American Network for Implementation and Validation of Pharmacogenomic Clinical Guidelines (RELIVAF-CYTED), Santiago 8500000, Chile; ismaelares@yahoo.com; 3 Pediatric Hemato-Oncology Service, State Cancer Center (CECAN), Durango 34000, Mexico; earedgo@yahoo.com (E.A.R.E.); lourdescordovah@hotmail.com (L.P.C.H.); 4 Pediatric Service, General Hospital of Durango, Maternal and Child Tower, Durango 34000, Mexico; aegfont49@gmail.com (A.E.G.F.); alonsogandaramireles@gmail.com (G.V.V.); 5 Epidemiology Service, State Cancer Center (CECAN), Durango 34000, Mexico; alfer.jp@gmail.com (C.M.M.); drpayangandara17@gmail.com (H.P.G.); 6 Faculty of Medicine and Psychology, Autonomous University of Baja California, Tijuana 22390, Mexico; leslie.patron@uabc.edu.mx (L.P.-R.); almanzareyes@hotmail.com (H.A.-R.)

**Keywords:** doxorubicin, cardiotoxicity, oxidative stress, 8-OHdG, pharmacogenetics, acute lymphoblastic leukemia, pediatric oncology

## Abstract

Doxorubicin (Dox) is a cornerstone in the treatment of pediatric acute lymphoblastic leukemia (ALL), but its use is limited by dose-dependent cardiotoxicity. Oxidative stress, arising from mitochondrial dysfunction, enzymatic generation of reactive oxygen species, and cardiotoxic metabolites, has been implicated as a central mechanism, with interindividual variability partly influenced by genetic factors. This study evaluated oxidative DNA damage 8-hydroxy-2′-deoxyguanosine (8-OHdG) as an integrative marker of redox-related pathways in Dox-induced cardiotoxicity. In a prospective case–control study, 93 pediatric patients with ALL treated with Dox and 63 controls were included. Cardiotoxicity was assessed by serial echocardiography, and 8-OHdG levels were measured by ELISA. Genotyping of *ABCC1* rs3743527, *NCF4* rs1883112, and *CBR3* rs1056892 was performed, and multivariable analyses were conducted. Dox-treated patients showed higher 8-OHdG levels than controls, and patients with cardiotoxicity (*n* = 11) had higher levels than those without. A higher frequency and severity of cardiotoxicity was observed in female patients, although this finding should be interpreted cautiously. Although allele frequencies did not reach statistical significance, distinct distribution patterns were observed between groups. These findings suggest that 8-OHdG may function as an integrative marker of redox dysfunction associated with Dox-induced cardiotoxicity.

## 1. Introduction

Childhood leukemia, especially acute lymphoblastic leukemia (ALL), currently represents one of the most prevalent malignancies in childhood. It is estimated to account for approximately 25% of all childhood cancers worldwide [1]. In Mexico, leukemia also remains a public health concern, with an incidence rate of about 4.6 cases per 100,000 children under 15 years of age [2]. ALL is characterized by the abnormal proliferation of immature cells called lymphoblasts, which would normally develop into lymphocytes, a type of white blood cell essential to the immune system. These lymphoblasts multiply rapidly and crowd out normal blood cells such as red blood cells and platelets, causing symptoms such as anemia, bruising, recurrent infections, and fatigue [3].

Standard treatment protocols for acute lymphoblastic leukemia (ALL) rely on combination chemotherapy regimens that include doxorubicin (Dox) [4]. As an anthracycline, Dox has demonstrated substantial efficacy in eradicating malignant cells; however, its clinical use is constrained by marked interindividual variability and the risk of cardiotoxicity [5,6]. Its primary mechanism of action is intercalation into cellular DNA, which means that Dox is physically inserted between the nitrogenous bases of DNA [7]. This intercalation disrupts the DNA structure and blocks the ability of cellular enzymes to read and copy the genetic sequence during cell replication, halting the synthesis of new DNA strands and ultimately inhibiting cell division and cancer cell proliferation [8]. Although this mechanism is not selective for leukemic cells, rapidly proliferating malignant cells are more susceptible to DNA damage induced by Dox.

**Figure 1:**
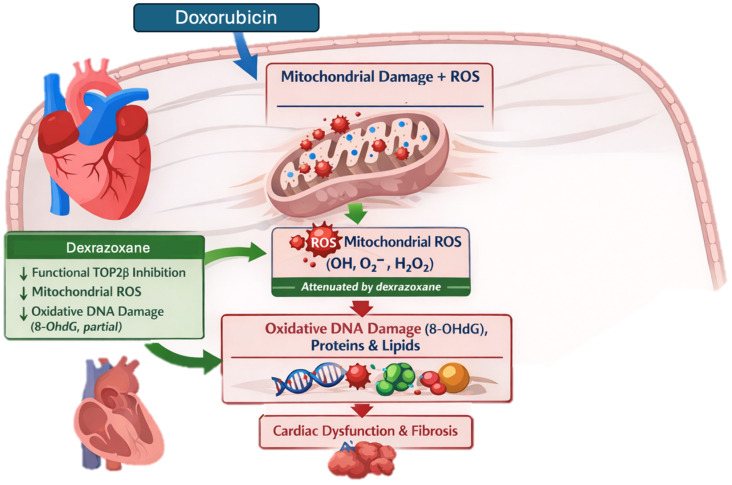
Proposed mechanism of Dox-induced oxidative stress and cardiotoxicity. Dox promotes mitochondrial dysfunction and reactive oxygen species (ROS) generation, leading to oxidative damage to DNA, proteins, and lipids, including the formation of 8-hydroxy-2′-deoxyguanosine (8-OHdG). Dexrazoxane attenuates oxidative injury and mitochondrial ROS production, although residual damage may contribute to cardiac dysfunction and fibrosis.

**Figure 2:**
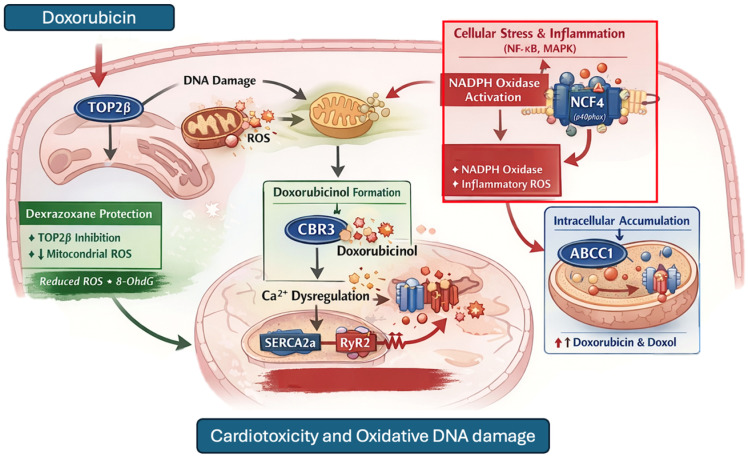
Proposed mechanisms involved in Dox-induced cardiotoxicity and oxidative DNA damage. Dox promotes TOP2β-mediated DNA damage and mitochondrial ROS generation. Additional oxidative stress may result from NADPH oxidase activation, influenced by *NCF4*, and from the formation of doxorubicinol mediated by *CBR3*. Intracellular accumulation of Dox and doxorubicinol may be modulated by *ABCC1*. These pathways contribute to calcium dysregulation through *SERCA2a* and *RyR2*, leading to cardiotoxicity and oxidative DNA damage. Dexrazoxane attenuates TOP2β-mediated injury and reduces ROS production, although residual oxidative stress may persist. Red arrows indicate pathways promoting oxidative injury, whereas green arrows indicate the protective effects of dexrazoxane.

In addition to its effect on DNA, Dox, as a result of its metabolism, also usually generates the production of reactive oxygen species (ROS) within cells [9,10]. During its intracellular metabolism, Dox generates free radicals such as superoxide anion (O_2_^−^) and hydrogen peroxide (H_2_O_2_) as by-products. These reactive oxygen species are highly reactive molecules containing oxygen with unpaired electrons in their structure, making them highly unstable and prone to unwanted chemical reactions within the cell [11,12]. Dox-induced oxidative stress is believed to arise from multiple mechanistically distinct pathways, in addition to mitochondrial dysfunction secondary to topoisomerase IIβ (TOP2β) mediated DNA damage, Dox can trigger redox cycling and iron-dependent reactions that amplify mitochondrial ROS production [13]. In parallel, cellular stress and inflammatory signaling activate enzymatic sources of ROS, such as NADPH oxidase, contributing to superoxide generation at the plasma membrane [14], and interindividual variability in components of the NADPH oxidase complex may therefore modulate the magnitude of enzymatic ROS generation in response to Dox exposure [15]. Moreover, metabolic conversion of Dox to doxorubicinol by carbonyl reductases (such as CBR3) promotes mitochondrial and calcium-handling dysfunction, which may indirectly increase oxidative stress over time [16,17,18]. Genetic variability in carbonyl reductases could influence the rate of doxorubicinol formation and, consequently, the extent of downstream mitochondrial dysfunction and oxidative stress [18]. Finally, intracellular exposure to Dox and its metabolites is modulated by ABC transporters (such as ABCC1), shaping the magnitude and persistence of oxidative injury [19]. Excessive ROS generation can be particularly detrimental to cardiac cells because the heart has a high metabolic demand and high lipid content, making it susceptible to oxidative stress [20,21]. Oxidative stress, defined as an imbalance between the production of reactive oxygen species (ROS) and the body’s antioxidant capacity to neutralize them, has emerged as a critical factor in a variety of diseases, including cardiovascular disease and cancer [22]. In this context, excessive generation of ROS during Dox metabolism could lead to oxidative damage to DNA, proteins, and lipids in cardiac cells, ultimately resulting in cardiac dysfunction and fibrosis (Figure 1) [10]. In this regard, Christidi E et al. [23]. point out that the primary source of cell damage leading to cardiomyocyte cell death is excessive ROS production, and Doroshow JH et al. [24], in their work aimed at examining the role of Dox-induced ROS in relation to cardiac apoptosis in cell lines, found that intracellular levels of Dox-induced ROS play an important role in Dox-induced apoptosis and altered cell cycle progression in murine cardiac fibroblasts. In this context, dexrazoxane is currently the only clinically approved cardioprotective agent for patients receiving anthracyclines. Dexrazoxane exerts its protective effects primarily by interfering with Dox topoisomerase IIβ complex formation in cardiomyocytes, thereby attenuating mitochondrial DNA damage and downstream mitochondrial ROS generation. However, dexrazoxane is not described as a broad-spectrum antioxidant nor as a direct inhibitor of all enzymatic or metabolic sources of oxidative stress, suggesting that oxidative injury may persist in a subset of patients despite cardioprotection (Figure 1 and Figure 2) [25,26].

Despite significant advances in the understanding of Dox-induced cardiotoxicity, several unknowns remain, especially regarding the specific role of oxidative stress and its potential as a therapeutic target. As mentioned above, DNA undergoes oxidative damage when exposed to ROS, such as free radicals, which can be produced as byproducts of cellular metabolism or in response to exogenous oxidizing agents such as the administration of Dox [7,8,9,10]. One of the products of this damage is 8-OHdG, a modified DNA base formed when guanosine (G) in DNA is oxidized by ROS [27]. By detecting and quantifying 8-OHdG in biological samples, it is possible to obtain information about the level of oxidative DNA damage in the organism [28]. This can be useful for the assessment of oxidative stress in various physiological and pathological conditions. This is mentioned by Di Minno A et al. [27], who in their work found that 8-OHdG levels were higher in patients with cardiovascular disease than in healthy patients; likewise, it also points out that 8-OHdG is generated after repair of ROS-mediated DNA damage and, therefore, is one of the most recognized biomarkers of oxidative DNA damage because guanosine is the most oxidized among the nucleobases. Thus, DNA damage has been shown to be significantly associated with the development of cardiovascular disease [29]. Importantly, while oxidative stress has been extensively implicated in anthracycline-induced cardiotoxicity, most studies have evaluated isolated mechanisms or single biomarkers, without integrating multiple oxidative pathways into a unified clinical-molecular framework. In this context, understanding how oxidative DNA damage reflects the convergence of mitochondrial dysfunction, enzymatic ROS production, and pharmacogenetic variability remains a critical gap. The aim of this study was to evaluate oxidative DNA damage (8-OHdG) as an integrative intermediate phenotype reflecting the convergence of oxidative stress-related molecular pathways in Dox-induced cardiotoxicity.

## 2. Materials and Methods

### 2.1. Study Design

This study was designed as a prospective, longitudinal case–control investigation incorporating complementary analytical components. Two distinct but related comparisons were performed: (1) an external comparison between pediatric ALL patients treated with doxorubicin and healthy controls to assess the overall burden of oxidative stress, and (2) an internal comparison within the ALL cohort (patients with and without cardiotoxicity) to identify factors associated with cardiotoxicity within a shared disease and treatment context. This dual analytical approach allows differentiation between general oxidative stress associated with disease and treatment, and interindividual variability related to susceptibility to cardiotoxicity. A total of 156 pediatric patients were included, 93 of them (case group) with a diagnosis of ALL who were being cared for at the Pediatric Haemato-Oncology service in the SSA Cancerology State Center (CECAN) in Durango, México, and 63 (control group) without any apparent disease who attended the clinical analysis laboratory of the General Hospital of Durango, Mexico. Of the ALL patients, 11 of them developed cardiotoxicity of any type. Rather than aiming to establish causal inference, this study was designed to identify clinically relevant associations within a biologically coherent framework, prioritizing internal comparisons to reduce variability related to disease status and treatment exposure.

All patients were diagnosed with ALL according to the classification of the French–American–British Association of Hematology [30]. This research was approved by the Research Ethics and Research Committees of the General Hospital of Durango, Mexico, with registration number 516/019 and 593/022, and by the Research Ethics Committee of the CECAN of the SSA, Durango, Mexico, with registration number 005, in accordance with the Declaration of Helsinki and the Mexican General Health Law. Each patient was under treatment with chemotherapy according to the St Jude TOTAL XV protocol [31]. The administered dose of Dox was 30 mg/m^2^ with an infusion duration of 1 h. In addition, all patients received dexrazoxane as a cardioprotective agent prior to Dox administration. Dexrazoxane use in this cohort was consistent with institutional clinical practice and national regulatory approval for pediatric patients. All patients’ parents were asked to provide written informed consent, in addition, children older than 7 years were also asked to provide assent.

Eligibility criteria were established to minimize potential confounding factors related to oxidative stress and cardiac dysfunction. Inclusion criteria for the ALL cohort were pediatric patients diagnosed with ALL to French–American–British classification criteria [30], receiving Dox-containing chemotherapy under the St Jude TOTAL XV protocol, with baseline normal cardiac function prior to anthracycline exposure (LVEF > 50%, FS > 28%, and E/A ratio between 1 and 2). Patients were required to have serial echocardiographic follow-up and available blood samples for biomarker and genetic analyses.

Exclusion criteria included previous exposure to anthracyclines or other known cardiotoxic drugs, congenital or acquired cardiovascular disease, chronic renal or hepatic dysfunction, active systemic inflammatory or infectious diseases at the time of sampling, and the presence of other malignant or chronic systemic disorders potentially associated with altered oxidative stress status.

For the control group, inclusion criteria consisted of pediatric individuals without apparent disease or known chronic medical conditions, recruited from the clinical laboratory service of the General Hospital of Durango, Mexico. Controls with evidence of acute infection, inflammatory conditions, cardiovascular disease, renal or hepatic dysfunction, or previous exposure to cardiotoxic medications were excluded.

### 2.2. Cardiotoxicity Assessment

A total of 93 patients with ALL underwent echocardiographic evaluation (EE) to assess cardiotoxicity. Baseline cardiac function was considered normal when left ventricular ejection fraction (LVEF) was >50%, fractional shortening (FS) >28%, and the ratio of diastolic filling (E/A) was between 1 and 2 prior to Dox treatment [32]. A second EE was performed one month after the first Dox dose, followed by subsequent evaluations at 4, 8, 18, 28, and 36 months after Dox administration. For patients in the surveillance phase, echocardiographic evaluation was conducted as part of routine follow-up after completion of treatment.

Cardiotoxicity was defined as a ≥10% decrease in left ventricular ejection fraction (LVEF) from baseline to a value below 50%, following Dox administration [33,34]. Once cardiotoxicity was identified, severity was classified according to absolute LVEF values as follows: severe (LVEF ≤ 30%), moderate (LVEF 31–40%), and mild (LVEF 41–50%). This classification was used to describe the spectrum of systolic dysfunction within the cohort. A decrease in FS below 28% after Dox administration was considered indicative of systolic dysfunction [35]. Diastolic dysfunction was defined as an altered E/A ratio (<1 or >2) [36].

### 2.3. Evaluation of Cardiovascular Risk Factors

The evaluation of cardiovascular risk factors at CECAN in Durango, Mexico currently takes into account high blood pressure, high cholesterol, overweight and obesity, smoking, physical inactivity, male gender, and age. None of the included participants had documented pre-existing cardiovascular disease or clinically identified cardiovascular risk conditions at the time of enrollment (except for gender, which was already taken into account in the association analysis).

### 2.4. Sample Collection

Blood samples were collected through an intravenous cannula previously inserted in the arm of each patient receiving chemotherapy. A total of 3 mL of blood sample was collected in plastic tubes with heparin as anticoagulant (Sarstedt^®^, Nümbrecht, Germany). Samples were stored at −80 °C until further analysis. Blood samples for 8-OHdG determination were collected prior to the administration of Dox and subsequently before each scheduled echocardiographic evaluation. Serial sampling was performed throughout follow-up (up to 36 months after treatment initiation or until the development of cardiotoxicity), allowing assessment of cumulative oxidative DNA damage over time rather than acute pharmacodynamic changes.

### 2.5. Genotyping

All patients were genotyped for single nucleotide variants (SNV) *ABCC1* rs3743527, *NCF4* rs1883112, and *CBR3* rs1056892, which are involved in key metabolic and redox-related pathways of Dox. DNA was obtained from whole blood using the “DTAB-CTAB” extraction procedure [37]. Its integrity and purity were determined by horizontal electrophoresis in 1% agarose gel, stained with Texas Red, and the quantification was carried out by spectrophotometry in Nanodrop^®^ (Thermo Scientific, Waltham, MA, USA). The SNV *ABCC1* rs3743527(C___8934057_30), *NCF4* rs1883112 (C__11521119_1_), and *CBR3* rs1056892 (C___9483603_10) were determined by real-time polymerase chain reaction (qPCR) using TaqMan technology in a specific thermocycler, Bios^®^StepOne^®^ (Foster City, CA, USA).

### 2.6. 8-OHdG Analysis

8-OHdG was quantified from DNA extracted from whole blood samples, reflecting systemic oxidative DNA damage rather than circulating free 8-OHdG levels. Genomic DNA was isolated using the DTAB–CTAB extraction method. DNA integrity and purity were assessed by horizontal electrophoresis on a 1% agarose gel stained with Texas Red, and concentrations were determined by spectrophotometry using a Nanodrop^®^ system (Thermo Scientific, Waltham, MA, USA). 8-OHdG levels were measured using a commercially available 8-hydroxy-2′-deoxyguanosine (8-OHdG) ELISA kit (Abcam, Cambridge, UK, 96-well format) [38], following the manufacturer’s instructions. Serial dilutions of both standards and samples were prepared and analyzed in triplicate. Absorbance was recorded at 450 nm. Given its biological role as a product of DNA repair following oxidative damage, 8-OHdG was interpreted as an integrative marker of cumulative oxidative stress rather than a pathway-specific indicator, allowing the capture of convergent molecular processes. The present study evaluated DNA-bound 8-OHdG derived from genomic DNA extracted from whole blood samples. Circulating free 8-OHdG levels were not assessed.

### 2.7. Statistical Analysis

Data are presented as mean ± standard deviation (SD) or as proportions, as appropriate. Differences between continuous variables were assessed using the Student’s *t*-test for independent samples, while categorical variables were analyzed using the chi-square test or Fisher’s exact test, as appropriate. Data dispersion was evaluated using TIBCO Statistica version 13.3 software [39,40], and density plots were generated using R software (version 4.6.0, R Foundation for Statistical Computing, Vienna, Austria) with the RStudio interface. Multivariable logistic regression models were constructed to evaluate the association between 8-OHdG levels and cardiotoxicity, as well as with doxorubicin exposure and genotype groups (*ABCC1* rs3743527, *NCF4* rs1883112, and *CBR3* rs1056892). Models were adjusted for clinically relevant covariates, including sex, cumulative doxorubicin dose, and genetic variants. Variable selection was guided by biological plausibility and prior evidence. Statistical significance was defined as a two-sided *p*-value < 0.05, with corresponding 95% confidence intervals (CIs). All statistical analyses were performed using PASW Statistics version 18.0.0 software. Due to the homogeneous administration of dexrazoxane across all patients, this variable was not included in regression models but was considered in the interpretation of results as a potential modulator of oxidative stress pathways.

## 3. Results

### 3.1. Study Population and Baseline Characteristics

The study included a total of 156 pediatric patients, of which 93 (cases) had a diagnosis of ALL and were being treated with Dox, and 63 (controls) had no apparent disease and were not receiving Dox. Table 1 shows the demographic characteristics of the patients, where a statistically significant difference was found for weight and BMI between groups (*p* = 0.04 and *p* = 0.04, respectively).

### 3.2. Oxidative DNA Damage Between Groups

**Figure 3:**
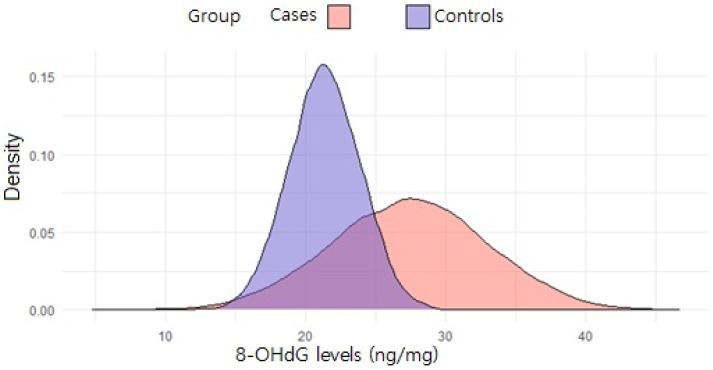
Distribution of 8-OHdG levels in cases and controls. The density plot shows the comparison of 8-OHdG levels in the case (pink color) and control (purple color) groups. The curve represents the number of patients at a given 8-OHdG level.

**Figure 4:**
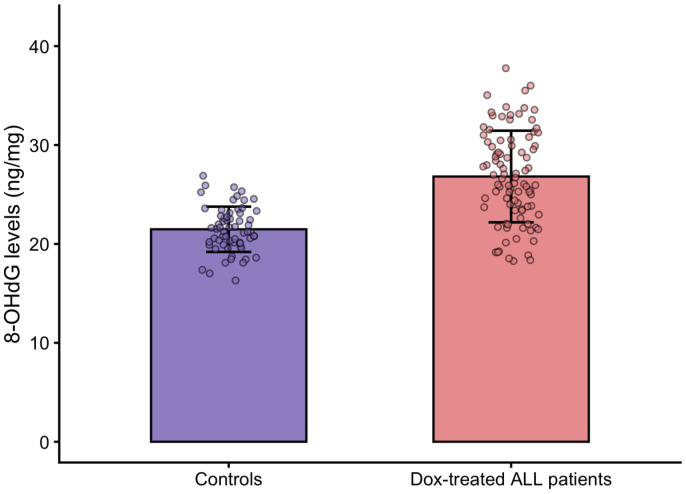
Bar graph showing mean 8-OHdG levels in controls and doxorubicin-treated ALL patients. Bars represent mean ± SD, and individual data points are overlaid to illustrate data distribution within each group. Doxorubicin-treated ALL patients exhibited significantly higher 8-OHdG levels compared with controls (*p* = 0.001).

All patients (cases and controls) had their 8-OHdG levels measured to determine whether there were differences in oxidative DNA damage between groups. Table 2 shows the differences in mean 8-OHdG levels, with significantly higher levels observed in patients with ALL receiving Dox compared to controls (*p* = 0.001). Figure 3 shows the density distribution of 8-OHdG levels in both groups, demonstrating a shift toward higher values in cases. Additionally, Figure 4 presents a bar graph with overlaid individual data points to facilitate direct visualization and comparison of 8-OHdG levels between controls and doxorubicin-treated ALL patients. Given the longitudinal design of biomarker collection, 8-OHdG levels reflect cumulative oxidative DNA damage across treatment phases rather than isolated measurements.

### 3.3. Association Between 8-OHdG and Cardiotoxicity

**Figure 5:**
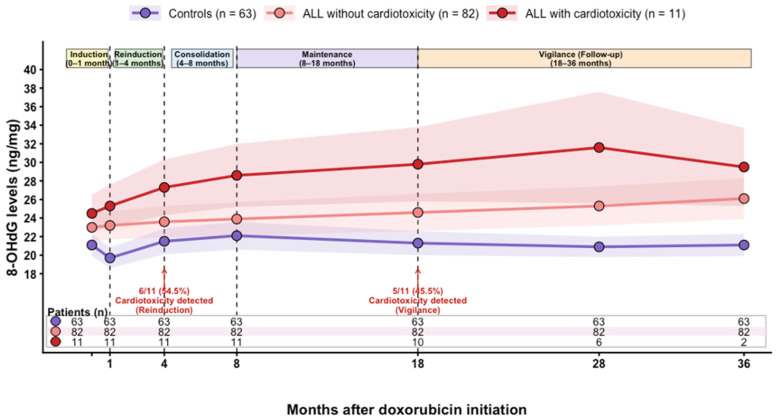
Longitudinal changes in 8-OHdG levels during treatment and follow-up in controls, ALL patients without cardiotoxicity, and ALL patients with cardiotoxicity. Lines represent mean values and shaded regions represent variability across serial measurements. Treatment phases are indicated at the top of the figure. Patients who developed cardiotoxicity showed persistently higher 8-OHdG levels throughout follow-up, with progressive increases during reinduction and vigilance phases. Arrows indicate the timing of cardiotoxicity detection events. The table below the graph shows the number of patients available at each time point.

Within the case group, 11 patients developed cardiotoxicity and 82 did not. Table 3 shows the comparison of mean 8-OHdG levels between patients who developed cardiotoxicity and those who did not. A statistically significant difference was observed, with higher 8-OHdG levels in patients who developed Dox-induced cardiotoxicity (31.56 ± 3.98 vs. 26.15 ± 2.56 ng/mg; t = 4.32; *p* = 0.03). These findings support an association between oxidative DNA damage and cardiotoxicity within a shared disease and treatment context, without implying a direct causal relationship. These findings are consistent with a model in which oxidative DNA damage reflects the downstream convergence of multiple molecular pathways involved in doxorubicin-induced cellular injury. Additionally, longitudinal assessment of 8-OHdG levels across treatment and follow-up phases demonstrated persistently higher oxidative DNA damage in patients who developed cardiotoxicity compared with both controls and ALL patients without cardiotoxicity (Figure 5). Progressive increases in 8-OHdG levels were particularly evident during late treatment and follow-up phases, coinciding with the temporal distribution of cardiotoxicity events. Specifically, 54.5% of cardiotoxicity cases were detected during reinduction therapy and 45.5% during the vigilance phase. In contrast, controls showed relatively stable 8-OHdG levels throughout follow-up, whereas ALL patients without cardiotoxicity exhibited a more gradual increase over time.

### 3.4. Pharmacogenetic Analysis

All patients were genotyped for SNVs ABCC1 rs3743527, NCF4 rs1883112, and CBR3 rs1056892. Table 4 shows the genotype and allele frequencies of these variants in patients with and without cardiotoxicity. Genotype distributions were in Hardy–Weinberg equilibrium. No statistically significant associations were observed between the analyzed SNVs and cardiotoxicity; however, differences in genotype distribution were observed between patients with and without cardiotoxicity.

### 3.5. Characterization of Cardiotoxicity Cases

Within the ALL cohort, cardiotoxicity cases were further characterized according to severity, treatment phase, cumulative Dox exposure, 8-OHdG levels, and genetic background. Table 5 shows the temporal distribution of cardiotoxicity findings according to LVEF values, as well as Dox doses and 8-OHdG levels.

Among the 93 patients, 11 developed cardiotoxicity: 8 were girls and 3 were boys. One patient had severe cardiotoxicity (LVEF ≤ 30%), 6 had moderate cardiotoxicity (LVEF 31–40%), and 4 had mild cardiotoxicity (LVEF 41–50%). All these patients were in post-remission phases (6 in reinduction therapy and 5 in vigilance). The average 8-OHdG concentration in patients with cardiotoxicity was 31.56 ± 3.98 ng/mg, higher than that observed in the overall case population.

Notably, the temporal pattern of cardiotoxicity and 8-OHdG levels appeared to differ according to genetic background. Among patients carrying CBR3 rs1056892 variant genotypes, 8 of 11 (72.7%) cardiotoxicity cases harbored this variant, and 5 of these 8 patients (62.5%) developed cardiotoxicity at later stages, predominantly during the vigilance phase, whereas 3 of 8 (37.5%) developed cardiotoxicity during reinduction therapy. These patients more frequently presented moderate to severe cardiotoxic phenotypes.

In contrast, NCF4 rs1883112 variant genotypes were observed in 7 of 11 patients (63.6%) with cardiotoxicity, of whom 6 of 7 (85.7%) developed cardiotoxicity earlier during reinduction/maintenance phases and 1 of 7 (14.3%) during vigilance, with predominantly mild-to-moderate clinical manifestations. These observations describe differences in temporal and severity patterns according to genetic background; however, these findings should be interpreted as descriptive patterns rather than statistically supported differences.

### 3.6. Relationship Between 8-OHdG, Cardiotoxicity Severity, and Sex

**Figure 6:**
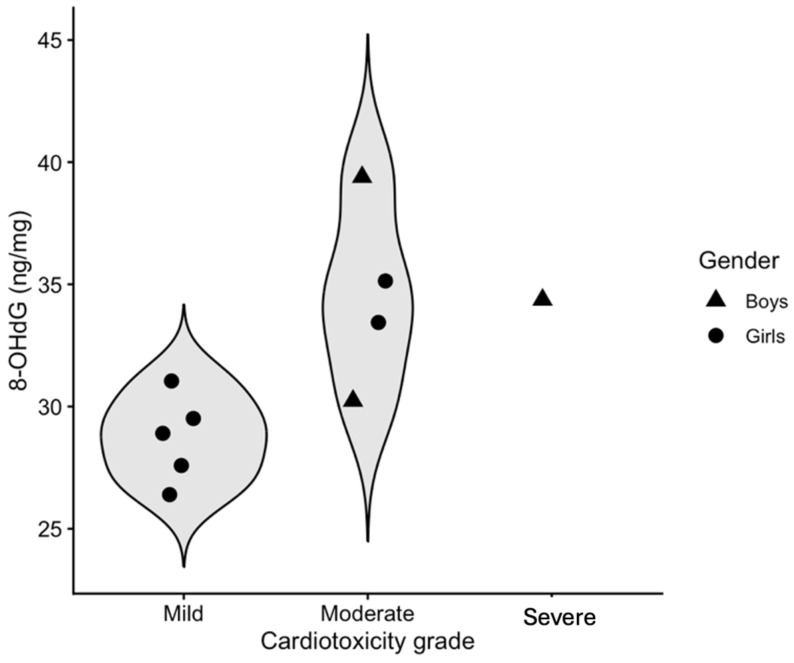
Violin plot with overlaid individual data points showing the distribution of 8-OHdG concentrations according to cardiotoxicity grade (mild, moderate, and severe). Each point represents a patient; the x-axis corresponds to cardiotoxicity grade and the y-axis to 8-OHdG concentration (ng/mg). Triangles represent boys and circles represent girls. The violin-shaped contours illustrate the distribution of values within each severity category.

Figure 6 shows the relationship between 8-OHdG concentration and cardiotoxicity severity, segmented by gender. No general trend was observed between 8-OHdG concentration and severity. While the overall trend was similar for both sexes, differences were observed in the distribution of cardiotoxicity severity, with more girls presenting moderate and mild toxicity compared to boys. Additionally, the only case of severe cardiotoxicity presented markedly elevated 8-OHdG levels.

### 3.7. Relationship Between 8-OHdG and Cumulative Dox Dose

**Figure 7:**
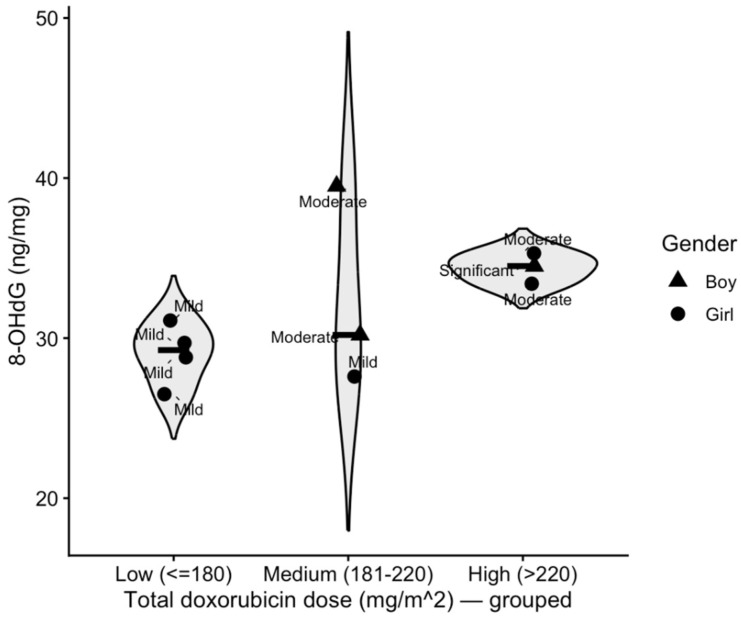
Relationship between 8-OHdG concentration levels with total patient doses. The dots represent individual patients differentiated by sex. The y-axis shows the 8-OHdG levels for each dot, and the x-axis shows the total Dox doses.

Figure 7 shows the relationship between 8-OHdG levels and cumulative Dox doses. An increasing trend was observed; however, this relationship did not appear to be strictly linear.

Of the 11 patients with cardiotoxicity, all met the predefined criteria based on LVEF reduction. Seven also had reduced FS (<28%) and four presented an altered E/A ratio (<1 or >2).

### 3.8. Echocardiographic Indicators and Oxidative Stress

Table 6 shows the comparison between echocardiographic indicators and their detection in patients with cardiotoxicity, along with average 8-OHdG levels. A statistically significant difference was observed between indicators, and the highest 8-OHdG levels were found in patients with altered LVEF.

### 3.9. Sex-Based Analysis

Table 7 shows the comparison between the number of patients with cardiotoxicity, mean 8-OHdG levels, and percentages of systolic and diastolic dysfunction between girls and boys.

Systolic dysfunction (LVEF reduction) was present in 100% of both girls (8/8) and boys (3/3), precluding statistical comparison. For diastolic dysfunction, 4 of 8 girls (50%) presented abnormalities, whereas none of the boys (0/3) did; however, this difference did not reach statistical significance (*p* = 0.20). Mean 8-OHdG levels were higher in girls (32.23 ± 2.2) than in boys (29.3 ± 1.7), although this difference did not reach statistical significance (*p* = 0.07).

Together, these findings suggest a possible sex-related trend toward greater oxidative stress burden and diastolic involvement in female patients; however, these findings should be interpreted as descriptive trends rather than statistically supported differences.

## 4. Discussion

The present study aimed to explore potential interactions between oxidative stress, genetic background, and clinical characteristics in the development of Dox-induced cardiotoxicity in pediatric patients with ALL, using 8-OHdG as an integrative intermediate phenotype. Importantly, the present study does not aim to isolate a single mechanistic pathway, but rather to provide an integrative perspective in which oxidative DNA damage reflects the convergence of multiple functional molecular processes, including mitochondrial dysfunction, enzymatic ROS generation, and pharmacogenetic modulation.

We found statistically significant differences between the groups for weight and BMI in the two groups (*p* = 0.04 and *p* = 0.04, respectively). Although weight and BMI differed between groups, these variables were not a primary focus of the present study and should be interpreted cautiously in relation to oxidative stress findings [41,42,43,44]. An important consideration in this study is the universal administration of dexrazoxane across all patients. Therefore, the observed associations may represent conservative estimates of oxidative injury and cardiotoxicity risk within a cardioprotected population. Dexrazoxane exerts its cardioprotective effects primarily through inhibition of TOP2β-mediated mitochondrial damage, thereby attenuating downstream oxidative stress. Consequently, its use may have influenced both the incidence of cardiotoxicity and the observed levels of oxidative DNA damage (8-OHdG), potentially acting as a confounding factor in the associations evaluated. Notwithstanding this, the persistence of cardiotoxicity and its association with oxidative DNA damage suggest that additional mechanisms beyond TOP2β-mediated injury may contribute to individual cardiac susceptibility.

To determine if there were differences in the levels of oxidative damage between the groups, patients in both groups were measured for 8-OHdG levels and a statistically significant difference was found between patients who had ALL and received Dox and those who did not have ALL and did not receive Dox (*p* = 0.001). Several authors have pointed out the use of 8-OHdG as an excellent candidate as a marker of oxidative stress generated in various diseases including cancer [45,46,47], as well as specifically in Dox-induced cardiotoxicity as mentioned by Li W et al. [48]. In this regard, Dulf PL et al. [49], in their work whose aim was to investigate the alterations of acute cardiotoxicity in the process of Dox-induced acute myocardial toxicity by investigating oxidative stress and autophagy markers, found that oxidative homeostasis was altered as early as 7 days after Dox treatment. This is consistent with our results, as we found that the group of cases that received Dox had a higher concentration of 8-OHdG than the healthy patients who did not receive Dox. Because ALL is intrinsically associated with systemic inflammation and increased oxidative stress, comparisons between ALL patients treated with doxorubicin and healthy controls must be interpreted within a broader biological context. Rather than serving as a direct comparator for treatment effects, the healthy control group was included to establish a physiological baseline of oxidative DNA damage. This approach allows interpretation of oxidative stress as a biological continuum, in which baseline levels observed in healthy individuals are amplified by disease-related processes in ALL, and further increased in the presence of treatment-associated cardiotoxicity.

Moreover, emerging evidence indicates that children with leukemia may exhibit subclinical cardiac functional alterations at diagnosis, prior to anthracycline exposure [50], further supporting the need to interpret oxidative stress within a disease-related framework. In this context, the internal comparison within the ALL cohort (patients with and without cardiotoxicity) represents a key analytical strength, as it enables the evaluation of interindividual variability in oxidative DNA damage within a shared disease and treatment environment.

Additionally, the absence of a comparator group not receiving dexrazoxane limits the ability to assess the independent effect of cardioprotection on oxidative stress and cardiotoxicity risk. However, given its homogeneous use across the cohort, the observed associations may reflect conservative estimates of oxidative injury within a cardioprotected population. Furthermore, cardiotoxicity severity in this study was classified based on absolute LVEF thresholds to describe the spectrum of systolic dysfunction within the cohort. However, this approach does not fully align with current ESC cardio-oncology guideline definitions [51], which are not strictly based on fixed LVEF categories. Although more sensitive modalities, such as myocardial strain imaging or cardiac biomarkers, were not available, LVEF-based criteria remain widely used in pediatric oncology practice, particularly in resource-limited settings. Accordingly, our approach reflects real-world clinical monitoring conditions.

The use of 8-OHdG as an intermediate phenotype provides a biologically plausible association between oxidative stress pathways and downstream clinical cardiotoxicity. Rather than reflecting a single ROS source, 8-OHdG integrates oxidative DNA damage arising from multiple convergent mechanisms, including mitochondrial ROS production, enzymatic ROS generation, and metabolic stress [22]. In this context, 8-OHdG may function as a systemic integrative marker of oxidative stress burden, capturing the cumulative impact of multiple convergent pathways rather than reflecting a single mechanistic source. The present study specifically focused on DNA-bound 8-OHdG derived from genomic DNA rather than circulating free 8-OHdG. This approach was selected because DNA-bound 8-OHdG reflects cumulative oxidative DNA damage at the cellular level and may better represent the integrated downstream effect of multiple redox-related pathways involved in Dox-induced injury. In contrast, circulating free 8-OHdG is generally considered to reflect systemic oxidative turnover and DNA repair activity. Accordingly, the present work was designed to evaluate oxidative DNA damage as an intermediate molecular phenotype associated with cardiotoxicity rather than as an acute circulating biomarker. The higher 8-OHdG levels observed in patients who developed cardiotoxicity, even within the same disease and treatment context, suggest its potential utility as a biomarker associated with susceptibility to anthracycline-induced cardiac injury. Beyond the overall association between 8-OHdG and Dox-induced cardiotoxicity, our results indicate that interindividual variability in oxidative stress pathways may contribute to heterogeneity in cardiotoxicity phenotypes. In particular, allele frequencies of *NCF4* rs1883112 and *CBR3* rs1056892 differed between patients with and without cardiotoxicity, suggesting a possible contribution of genetic modulation of enzymatic ROS generation (NADPH oxidase-related) and intracellular drug exposure, respectively. These observations suggest a potential pattern in the temporal and phenotypic expression of cardiotoxicity according to genetic background, although these findings were not statistically significant and should be considered exploratory. This pattern may reflect the involvement of distinct oxidative stress pathways in cardiac injury, which could differ in their clinical expression. Importantly, the longitudinal sampling design strengthens the interpretation of 8-OHdG as a cumulative marker of oxidative DNA damage over time. By collecting samples prior to Dox exposure and before each echocardiographic evaluation, this study captures temporal changes in oxidative stress throughout treatment and follow-up rather than relying on a single time-point measurement. The longitudinal analysis incorporated in the present study demonstrated persistently elevated 8-OHdG levels in patients who developed cardiotoxicity throughout treatment and follow-up. Progressive increases in oxidative DNA damage were particularly evident during maintenance and vigilance phases, coinciding with the temporal distribution of cardiotoxicity events. Although the relatively small number of cardiotoxicity cases limits definitive temporal inference, these findings support the hypothesis that sustained oxidative DNA damage accompanies the evolution of Dox-induced cardiac dysfunction over time. It is important to emphasize that 8-OHdG does not enable discrimination between distinct sources of reactive oxygen species and instead reflects a systemic oxidative stress burden rather than tissue-specific damage. Accordingly, the observed relationship between 8-OHdG levels and cardiotoxicity should be interpreted as an association rather than a direct mechanistic or causal link, and 8-OHdG should not be considered a cardiac-specific biomarker. Given the longitudinal yet observational design of the study, it is not possible to determine whether elevated 8-OHdG levels are predictive of cardiotoxicity or represent a downstream consequence of oxidative injury. Nevertheless, the observed association within a shared disease and treatment context supports the hypothesis that oxidative DNA damage may be linked to individual susceptibility to cardiotoxicity.

Similarly, within the group of cases, we found that 11 patients developed some type of cardiotoxicity, 8 of which were girls and 3 were boys; in addition, all children with cardiotoxicity had altered LVEF, 7 also had altered FS, and only 4 had altered E/A. With respect to gender, considering LVEF as the main marker of systolic damage and E/A as the main marker of diastolic damage [52], we found that all the girls (8) had altered LVEF and all the boys also had altered LVEF; in addition, 4 of the 8 girls also had an altered E/A ratio, but none of the boys had an altered E/A ratio. A trend toward higher frequency and severity of cardiotoxicity was observed in female patients; however, this difference did not reach statistical significance, in addition to a trend between systolic and diastolic toxicity, since none of the boys presented diastolic toxicity and 4 girls presented diastolic toxicity. These findings suggest a possible trend toward greater cardiotoxicity burden in female patients. Potential sex-related differences in cardiac susceptibility in pediatric patients may reflect differences in cardiac size and developmental factors, as well as hormonal and molecular influences [53]; there is greater damage to the heart in the event of contraction (systole) than in filling (diastole), however, the underlying mechanisms remain unclear and should be interpreted within the context of the study design. Hence, Canale ML et al. [54] highlight the fact that the risk of cardiotoxicity in girls who survived childhood cancer is about four times higher than in male survivors of childhood cancer treated with anthracyclines. Similarly, Lipshultz et al. [55] reported that the left ventricular contractility of female survivors of childhood cancer was significantly worse than that of their male counterparts after completion of Dox treatment. This makes sense with what we have found, since of the 11 patients with cardiotoxicity, all of them were post-remission induction (6 in Reinduction therapy and 5 in Vigilance).

The differential timing observed according to genetic background may reflect potential differences, although these observations are exploratory and require validation. Carriers of *CBR3* rs1056892 variant genotypes more frequently developed cardiotoxicity at later stages of treatment, which is biologically plausible given the role of *CBR3* in the metabolic conversion of DOX to doxorubicinol, a metabolite associated with progressive mitochondrial and calcium-handling dysfunction [8]. In contrast, *NCF4* rs1883112 variant carriers more often exhibited earlier cardiotoxicity with milder phenotypes, consistent with the involvement of NADPH oxidase-related enzymatic ROS generation in early oxidative signaling and injury [56]. Together, these observations are consistent with the possibility that distinct oxidative pathways may contribute to differences in the onset and severity of anthracycline cardiotoxicity, although these findings should be considered exploratory.

This study has limitations. First, the relatively small number of cardiotoxicity cases limits statistical power and precludes definitive conclusions regarding individual associations. However, the consistency of observed patterns within a longitudinal and internally controlled cohort supports their biological plausibility. Second, the observational design does not allow causal inference, and findings should be interpreted as hypothesis-generating. Third, 8-OHdG represents a systemic marker of oxidative DNA damage and does not provide tissue-specific resolution. Nevertheless, its integrative nature may be advantageous for capturing the cumulative effect of multiple converging molecular pathways.

Additionally, circulating free 8-OHdG levels were not evaluated in the present study. Although circulating 8-OHdG may provide complementary information regarding systemic oxidative stress burden and potential clinical biomarker applicability, the present work specifically focused on DNA-bound 8-OHdG as a marker of cumulative oxidative DNA damage. Future studies integrating both circulating and DNA-bound 8-OHdG measurements may help clarify their comparative clinical utility in Dox-induced cardiotoxicity.

Although external validation was not available, the internal consistency of the findings and their alignment with known biological mechanisms support their relevance as a framework for future mechanistic and multicenter studies.

## 5. Conclusions

This study provides exploratory evidence that oxidative DNA damage, assessed through 8-OHdG, may reflect redox dysfunction associated with doxorubicin-induced cardiotoxicity. These findings support a model in which multiple molecular processes converge into a systemic oxidative phenotype, contributing to interindividual variability in cardiotoxicity susceptibility.

## Figures and Tables

**Table 1:** Description of patient demographics (cases and controls), with and without doxorubicin treatment.

Variables	Cases *n* = 93	Controls *n* = 63	*p* *
Age (years)	10.6 ± 4.5	11.4 ± 4.6	0.24
Sex (M/F)	31/62	23/40	-
Weight (Kg)	31.9 ± 9.8	33.9 ± 8.8	0.04
Height (cm)	137.7 ± 24.7	135.3 ±19.2	0.09
BMI (kg/m^2^)	17.05 ± 1.8	18.66 ± 2.2	0.04

* Student’s *t*-test.

**Table 2:** 8-OHdG levels found in cases and controls with and without doxorubicin treatment, respectively.

Indicator	Cases (*n* = 93)	Controls (*n* = 63)	*p* *
8-OHdG	27.38 ± 5.63	21.34 ± 2.56	0.001

* Student’s *t*-test = 4.26.

**Table 3:** 8-OHdG levels found in cases with and without cardiotoxicity.

Indicator	With (*n* = 11)	Without (*n* = 82)	*p* *
8-OHdG	31.56 ± 3.98	26.15 ± 2.56	0.03

* Student’s *t*-test = 4.32.

**Table 4:** Genotype and allele frequencies of these variants in patients with and without cardiotoxicity.

Gene (SNV)	Genotype/Allele	Cases with Cardiotoxicity *n* = 11 (%)	Cases Without Cardiotoxicity *n* = 82 *n* (%)	*p*-Value	Hardy-Weinberg Equilibrium
*NCF4* rs1883112	WT (GG)	4 (36.4)	40 (48.8)	0.06	Yes
	HT (GA)	3 (27.2)	39 (47.6)		
	HM (AA)	4 (36.4)	3 (3.6)		
	Major allele (G)	11 (50.0)	119 (72.5)	0.16	
	Minor allele (A)	11 (50.0)	45 (27.5)		
*CBR3* rs1056892	WT (GG)	4 (36.4)	45 (54.9)	0.08	Yes
	HT (AG)	2 (18.1)	33 (40.3)		
	HM (AA)	5 (45.5)	4 (4.8)		
	Major allele (G)	10 (45.5)	123 (75.0)	0.10	
	Minor allele (A)	12 (54.5)	41 (25.0)		
*ABCC1* rs3743527	WT (CC)	7 (63.6)	36 (43.9)	0.10	Yes
	HT (CT)	3 (27.3)	32 (39.0)		
	HM (TT)	1 (9.1)	14 (17.1)		
	Major allele (C)	17 (77.3)	104 (63.4)	0.10	
	Minor allele (T)	5 (22.7)	60 (36.6)		

*n* = 93 participants included; *p*: statistical significance was established at *p* < 0.05; WT: wild type; HT: heterozygous; HM: homozygous mutant.

**Table 5:** Description of the temporal distribution of the cardiotoxicity findings, the dose of Dox, SNVs, and the 8-OHdG levels.

Number of Patients with Cardiotoxicity/Sex	Classification of Cardiotoxicity According to Severity			Temporal Distribution			[Dox] Final Accumulated	m^2^ Body Surface	[Dox] Total Amount	8-OHdG	SNVs		
	Severe Cardiotoxicity ^a^	Moderate Cardiotoxicity ^b^	Mild Cardiotoxicity ^c^	Remission Induction	Reinduction Therapy (Maintenance)	Vigilance	[ATC] *: High Risk ^d^/Low Risk ^e^		Dependent on the Surface Area of the Body.	[8-OHDG] (ng/mg)	*ABCC*13743527	*CBR3*rs1056892	*NCF4*rs1883112
1/M		X			X		180 mg/m^2^	1.15	203 mg/m^2^	30.2	HW	HM	HT
2/F			X		X		180 mg/m^2^	0.91	172 mg/m^2^	29.7	HW	HT	HT
4/M		X			X		180 mg/m^2^	1.05	203 mg/m^2^	39.5	HT	HW	HM
6/F		X				X	180 mg/m^2^	1.10	172 mg/m^2^	27.9	HW	HM	HW
9/F			X		X		180 mg/m^2^	0.99	188 mg/m^2^	31.1	HW	HM	HM
10/F		X				X	180 mg/m^2^	1.49	250 mg/m^2^	26.5	HW	HM	HW
11/M	X					X	180 mg/m^2^	1.32	237 mg/m^2^	28.8	HW	HM	HW
13/F			X		X		180 mg/m^2^	1.46	157 mg/m^2^	35.3	HW	HW	HM
15/F		X				X	180 mg/m^2^	1.27	227 mg/m^2^	33.4	HT	HM	HM
21/F			X		X		180 mg/m^2^	0.91	172 mg/m^2^	34.5	HT	HW	HT
33/F		X				X	180 mg/m^2^	1.46	157 mg/m^2^	30.1	HM	HM	HW
Promedio										31.56 ± 3.98			

^a^: LVEF ≤ 30%; ^b^: LVEF between 31% and 40%; ^c^: LVEF between 41% and 50%; ^d^ = 230 mg/m^2^ (50 mg of Dau and 180 of Dox); ^e^ = 150 mg/m^2^ (50 mg of Dau and 60 of Dox). [ATC] *: Anthracycline concentration.

**Table 6:** Echocardiographic abnormalities among patients with cardiotoxicity and corresponding 8-OHdG levels.

Ecocardiographic Indicators	Patients with Abnormal Finding	Patients Without Abnormal Finding	Patients	*p* *	8-OHdG
LVEF	11/11 (100%)	0/11 (0%)	11	0.01	32.4 ± 2.2
FS	7/11 (63.6%)	4/11 (36.4%)			29.8 ± 1.8
E/A	4/11 (36.4%)	7/11 (63.6%)			31.1 ± 1.6

8-OHdG values correspond to patients with abnormal findings for each echocardiographic parameter. * Chi square test. X2 = 8.57. LVEF = left ventricular ejection fraction; FS: fractional shortening; E/A = diastolic filling volume.

**Table 7:** Comparison between the number of patients with cardiotoxicity by sex, mean 8-OHdG levels, and the percentages of systolic and diastolic damage (considering LVEF and E/A ratio, respectively) between girls and boys.

Total	Girls *n* = 8 *n*/%	Boys *n* = 3 *n*/%	*p* *	[8OHdG]
Systolic Damage (LVEF)	08/100	3/100	0.2	32.4 ± 2.2
Diastolic Damage (E/A)	04/50	0/0		31.1 ± 1.6
[8OHdG]	32.23 ± 2.2	29.3 ± 1.7	0.07	

* Fisher’s exact test. LVEF = left ventricular ejection fraction. E/A = diastolic filling volume.

## Data Availability

The data presented in this study are available on reasonable request from the corresponding author. The data are not publicly available due to ethical and privacy restrictions intended to protect the confidentiality of pediatric patients and their sensitive clinical information. Access to the data may be granted following approval by the corresponding author and the relevant institutional ethics committees.

## Abbreviations

ALL	Acute lymphoblastic leukemia
Dox	Doxorubicin
ROS	Reactive oxygen species
8-OHdG	8-hydroxy-2′-deoxyguanosine
LVEF	Left ventricular ejection fraction
FS	Fractional shortening
E/A	Diastolic filling ratio
SNV	Single nucleotide variant
TOP2β	Topoisomerase II beta
CECAN	State Cancer Center
